# Using Pulsed Radiofrequency for Chronic Pain

**DOI:** 10.5812/kowsar.22287523.4047

**Published:** 2012-01-01

**Authors:** Farnad Imani

**Affiliations:** 1 Department of Anesthesiology and Pain Medicine, Rasoul-Akram Medical Center, Tehran University of Medical Sciences (TUMS), Tehran, Iran

**Keywords:** Pulsed Radiofrequency Treatment, Chronic Pain, Amputation

Radiofrequency thermocoagulation (RFTC) is a minimally invasive and target-selective modality procedure that has been used for over three decades. This has been demonstrated to be successful for reducing pain in the treatment of various chronic pain syndromes. Currently case reports and retrospective analysis of patient series suggest that pulsed radiofrequency (PRF) may be considered for the management of shoulder pain, glossopharyngeal neuralgia, head and facial pain, groin pain, meralgia paresthesia, and various types of neuropathic pain (1).

RFTC is a palliative treatment not without adverse effects. It has been reported to be associated with complications when compared with other ablative neurosurgical methods. Furthermore, conventional (continuous) radio frequency (RF) therapy sometimes results in a worsening and even the onset of new pain.

PRF is a non- or minimally neuroablative approach for various chronic pain conditions and thus is a less painful technique, it serves as an alternative to conventional RF treatment. It is used with the advantages of safe, easy application, and less adverse effects, compared to conventional RF therapy (2). The use of PRF promises to be a non-invasive and non-destructive approach for various chronic pain syndromes. The exact mechanism of its effect is not completely understood, but it is thought to be a neuromodulatory effect resulting from a pulsed electric field that might interfere with sensory neuron-specific gene expression and the molecules involved in the sensitization and development of neuropathic pain (3). The direct effect of the electrical field on the dorsal root ganglia (DRG) is a plausible explanation for inducing changes in the dorsal horn neurons.

Another theory postulates that the electrical fields reversibly disrupt the transmission of impulses across small un-myelinated neurons without damaging them completely, while the larger neurons remain protected by the myelin sheath and are thus unaffected (4-6).

Furthermore, since PRF does not produce a high enough temperature to damage the neural structures around the probe or the tissue, there is no risk of deafferentation pain after PRF application (7).

Degenerative cervical facet joint pain is, however, an important population condition commonly seen in the pain clinic. Radicular pain presumably originates in the DRG. In parallel with the positive findings of PRF adjacent to the cervical DRG for the management of radicular pain, well-designed random controlled trials (RCT) should shed light on the effect of PRF adjacent to the lumbar DRG for the management of lumbar radicular pain. Studies should concentrate on the effects of PRF treatment on neck pain due to degenerative facet joints. RF and PRF treatment may offer pain relief for patients suffering chronic pain refractory to conventional treatment. The currently available evidence should be complemented with well-designed trials. Study protocols should be designed to include selected patient populations. Attention should be paid to the inclusion criteria reflecting the “best available” diagnostic tests. The study protocol should be carefully designed to allow inclusion of well-selected patients. The tests used for patient inclusion in such a trial could potentially help the clinician in selecting patients for this type of treatment (1).

The evidence on PRF treatment of the peripheral nerves is scarce (8). The lower neurodestructive characteristics of PRF compared with RF may offer an alternative selective treatment approach (1). Although the observational studies report the clinical efficacy of PRF, the controlled clinical data on PRF is limited and provides a level-3 (C) evidence of its efficacy; support by one RCT or inconsistent findings in multiple RCTs (9). Despite the weakness of the controlled clinical data supporting its use, the apparent lack of side-effects and the wider applicability of PRF calls for further RCTs in order for the practicing pain physician to clearly understand its role in the treatment of various chronic pain syndromes (9).

In order to emphasize the importance of PRF in the treatment of chronic pain, we have published three reports in this issue (10-12). The authors of these articles reported successful applications of PRF for the treatment of pain conditions, including amputation pain, lumbar facet joint pain, and whiplash pain. These encouraging results need to be confirmed in well-designed RCTs.
